# Monocytes and neutrophils as ‘bad guys’ for the outcome of interleukin-2 with and without histamine in metastatic renal cell carcinoma – results from a randomised phase II trial

**DOI:** 10.1038/sj.bjc.6602937

**Published:** 2006-01-24

**Authors:** F Donskov, M Hokland, N Marcussen, H H Torp Madsen, H von der Maase

**Affiliations:** 1 Department of Oncology, Aarhus University Hospital, Denmark; 2 Department of Medical Microbiology and Immunology, University of Aarhus, Denmark; 3 Department of Pathology, Aarhus University Hospital, Denmark; 4 Department of Radiology, Aarhus University Hospital, Denmark

**Keywords:** renal cell carcinoma, interleukin-2, histamine dihydrochloride, oxidative stress

## Abstract

Histamine (HDC) inhibits formation and release of phagocyte-derived reactive oxygen species, and thereby protects natural killer (NK) and T cells against oxidative damage. Thus, the addition of histamine may potentially improve the efficacy of interleukin-2 (IL-2). We have explored this potential mechanism clinically in two randomised phase II trials in metastatic renal cell carcinoma (mRCC). In parallel with the clinical trial in Denmark (*n*=63), we obtained serial blood samples and tumour biopsies searching for a potential histamine effect *in situ*. At baseline and on-treatment weeks 3 and 8, we monitored the ‘good guys’ (i.e. NK and T cells) and ‘bad guys’ (i.e. monocytes/macrophages and neutrophils) simultaneously in blood (*n*=59) and tumour tissue (*n*=44). Patients with high number of monocytes and neutrophils in peripheral blood had very poor survival, with apparently no benefit from either IL-2 alone or IL-2/HDC treatment. Blood monocytes (*r*=−0.36, *P*=0.01) and neutrophils (*r*=−0.46, *P*=0.001) were negatively correlated with cytotoxicity, whereas blood NK cells were positively correlated with cytotoxicity (*r*=0.39, *P*=0.002). Treatment with IL-2 alone resulted in a significantly higher number of circulating monocytes (*P*=0.037) and intratumoral macrophages (*P*=0.005) compared with baseline. In contrast, IL-2/HDC resulted in an unchanged number of circulating monocytes and intratumoral macrophages, and in addition, a significantly increased number of intratumoral CD56^+^ NK cells (*P*=0.008) and CD8^+^ T cells (*P*=0.019) compared with baseline. The study provides evidence that circulating monocytes and neutrophils are powerful negative prognostic factors for IL-2-based immunotherapy and establishes a biological rationale for the potential use of histamine in conjunction with IL-2 in mRCC.

The observation that many cancers in adults arise in the setting of chronic inflammation has opened up a research field that has been crucial for our understanding and ultimately has established a new paradigm (Vakkila and Lotze, 2004). Solid tumours are not just composed of malignant cells (Hanahan and Weinberg, 2000), but are complex tissues in which tumour cells have attracted and educated tumour-infiltrating leucocytes (especially macrophages, neutrophils and mast cells) to serve as active collaborators (Coussens and Werb, 2001; Murdoch *et al*, 2004; Pollard, 2004). Through the production of growth factors, proteases, angiogenic mediators and reactive oxygen species (ROS), tumour-educated macrophages promote tumour growth, angiogenesis, metastasis and genomic instability (Balkwill and Mantovani, 2001; Coussens and Werb, 2002; Lin and Pollard, 2004; Wyckoff *et al*, 2004; Chen *et al*, 2005). Moreover, monocyte/macrophage-generated ROS are highly toxic to antitumour lymphocytes such as natural killer (NK) cells and T cells with the result that these cells undergo apoptosis (Hansson *et al*, 1996; Kono *et al*, 1996; Malmberg, 2004).

Monocytes/macrophages and neutrophils therefore represent an important drug-target for cancer treatment, with the aim of reducing the number and/or function of these cells. Extensive laboratory work for a decennium has identified histamine dihydrochloride (HDC) as an anti-phagocyte drug-candidate (Hellstrand, 2002). Targeting NADPH-oxidase through binding to the H_2_-receptor on monocytes (Hellstrand and Hermodsson, 1986) and neutrophils (Betten *et al*, 2003), HDC specifically blocks the formation and release of hydrogen peroxide (H_2_O_2_), thereby protecting NK and T cells from oxygen radical-induced inhibition and apoptosis (Hellstrand *et al*, 1994). Thus, NK and T cells remain viable and responsive to interleukin-2 (IL-2) (Hellstrand *et al*, 1990; Hellstrand and Hermodsson, 1990; Asea *et al*, 1996). IL-2 has no direct impact on tumour cells (Rosenberg, 2001) but requires NK and T-cells for tumourlysis (Donskov *et al*, 2002a, 2004a, 2004b). Thus, the addition of histamine may potentially improve the efficacy of IL-2.

We have explored this potential mechanism clinically in a randomised phase II trial in metastatic renal cell carcinoma (mRCC) (Donskov *et al*, 2005). As a supplement to the clinical trial in Denmark, the present biological study was initiated to search for a potential histamine effect *in situ*.

## MATERIALS AND METHODS

### Patients and treatment

Two randomised phase II trials of IL-2 with and without HDC were run in parallel, one in Aarhus, Denmark and one in Manchester, United Kingdom (Donskov *et al*, 2005). Only patients randomised in the Danish study were included in the blood and tumour study. The local ethics committee approved the study. All subjects gave written informed consent before blood samples and tumour biopsies. Study design, data accrual, data analyses and manuscript preparation were performed entirely by the authors.

In order to be able to correlate the baseline and on-treatment blood and tumour parameters with the *biological* effect of IL-2 with and without histamine, it was predefined that only patients receiving at least 80% of the scheduled drug dose within the first treatment course were assessable for the present analyses.

Patients were consecutively randomised to receive either IL-2/HDC or IL-2 alone. One cycle consisted of IL-2 (Aldesleukin, rIL-2, Proleukin®, Chiron, The Netherlands) as a fixed dose, 18 MIU s.c. once daily, 5 days per week for 3 weeks followed by 2 weeks rest. Histamine dihydrochloride (HDC, Ceplene™, supplied by Maxim Pharmaceuticals Inc., San Diego, USA), 1.0 mg was added twice daily, concomitantly with IL-2. Patients were evaluated for objective response, according to standard WHO criteria (Miller *et al*, 1981), every two cycles (10 weeks). A maximum of four treatment cycles was given.

### Tumour samples

Core needle biopsies (18 G cutting needle) were collected by standard ultrasound-guided procedures before treatment (baseline) and at day 2 in weeks 3 and 8. The week 3 and week 8 time points were selected to coincide with routine outpatient visit, according to the immunotherapy schedule. A total of 98 biopsies were obtained (Table 1) at baseline from 44 patients (IL-2/HDC, *n*=21; IL-2, *n*=23), at week 3 from 29 patients (IL-2/HDC, *n*=14; IL-2, *n*=15) and at week 8 from 25 patients (IL-2/HDC, *n*=12; IL-2, *n*=13). Based on well-known prognostic factors of MSKCC (Motzer *et al*, 1999), there were no significant differences between the baseline patient group and the week 3 or the week 8 group, *P*=0.8 and *P*=0.15, respectively, (Fisher's exact test). There was no significant difference between tumour histology types (IL-2/HDC, clear cell RCC 82%; IL-2, clear cell RCC 88%) (*P*=0.49).

Biopsies were performed from accessible tumour locations: kidney, *n*=42; liver, *n*=17; lung/pleura/chest wall, *n*=13; abdominal/pelvic soft tissue, *n*=10; lymph node, *n*=9; subcutis, *n*=7; kidney bed, *n*=6; and muscle, *n*=2; some patients had biopsies from more than one location. On-treatment biopsies were obtained from the same tumour as the baseline biopsy. Unfortunately, it showed up that none of the subsequent CR patients had accessible baseline tumours for core needle biopsies. Thus, these CR patients were not included in the statistical analyses for the tumour variables but were, however, included in the statistical analyses for the blood variables.

### Immunohistochemistry

Immunohistochemistry used standard procedures, as described in our previous studies (Donskov *et al*, 2002a, 2004a). Paraffin sections were stained with monoclonal antibodies from DakoCytomation, Denmark, CD8 (M7103 1 : 100), CD20 (M0755 1 : 500); NovoCastra, Denmark, CD4 (NCL-CD4-1F6 1 : 50), CD56 (NCL-CD56-1B6 1 : 40), MACRO (NCL-MACRO 1 : 80); Pharmingen, Denmark, CD57 (33251A 1 : 500), CD66b (33731A 1 : 100) and Ramcon, Denmark, zeta (IM2549 1 : 20). Primary antibodies were detected using the Envision-peroxidase system (K4000, DakoCytomation). All staining was performed in a TechMate automatic immunohistochemistry staining machine, (DakoCytomation, Denmark).

### Immunohistochemical evaluation

Intratumoural immune cells were counted by an objective and reproducible stereological method (Gundersen *et al*, 1988). A computer-generated unbiased counting frame was used to perform measurements. The first field of vision was chosen at random, after which the computer systematically sampled the subsequent fields of vision within the entire tumour biopsy. Necrosis, artefacts and fibrous areas were omitted. Using a × 40 objective, a total number of 40 fields (4951 *μ*m^2^ each) were counted, if the size of the tumour biopsy allowed for it. Only cells showing specific surface staining, a visible nucleus and location within the counting frame were counted as positive. The mean number of cells mm^−2^ tumour tissue was assessed for each patient. Staining was analysed blinded by one observer. Selected sections were counted blinded by a second experienced observer (NM) and a high level of reproducibility was demonstrated, as previously reported (Donskov *et al*, 2004a).

### Blood samples

Peripheral blood mononuclear cells were obtained (Table 1) and isolated from lithium-heparinized whole blood samples by Ficoll–Paque (Pharmacia Biotech, Uppsala, Sweden) gradient separation, washed twice and cryopreserved at −135°C until use. Cytolytic activity was determined by standard 4-h ^51^Cr-release assay against K562 target, as previously reported in detail (Donskov *et al*, 2002a). Differential blood cell counts were determined by standard Coulter counter technique (Coulter STKS) in the clinical chemistry laboratory.

Cell surface phenotypes were determined by flow cytometry using a Coulter XL-2 flow cytometer (Coulter Electronics, Florida). Data were analysed using the Flow-Jo software (Treestar, San Carlos, CA). Direct fluorochrome-conjugated antibodies (FITC or PE) were purchased from DakoCytomation, Denmark (CD3, CD4, CD8, CD20) and Becton Dickinson, Denmark (CD16, CD56, CD57, CD69). Intracellular zeta expression was investigated on permeabilised cells, using 2H2D9 (TIA-2) PE-conjugated MoAb (Ramcon, Denmark). Relevant isotype controls were purchased from DakoCytomation.

### Statistics

Overall survival time was measured from the first day of treatment until death or last follow-up evaluation. The relationship between assessed parameters and treatment arm was evaluated using the nonparametric Mann–Whitney *U*-test. The significance of changes from baseline to week 3 or to week 8 was assessed using the Wilcoxon signed rank test for paired samples. Differences in patient characteristics and response rates were analysed by Fisher's exact test. The cumulated survival rate was analysed by Kaplan–Meier and the log-rank test was used to analyse for survival differences among subgroups of patients. The median follow-up period was 43 months (range 32–51 months). No patients were lost to follow-up. Data were updated April 14, 2005. Statistical analyses were performed using SPSS v11.0.

## RESULTS

### Overall clinical outcome

Between August 2000 and August 2002, 63 patients with mRCC were enrolled in a Danish single-centre randomised phase II trial. Thirty-three patients were randomly assigned to IL-2/HDC therapy and 30 to IL-2 alone. The clinical outcome of this and a parallel UK study has been published separately (Donskov *et al*, 2005). In summary, our Danish study showed a statistically significant 1-year survival benefit (76 *vs* 47%, *P*=0.03) and a trend towards an improved median survival time (18.3 *vs* 11.4 months, *P*=0.07) and an improved clinical benefit (CR+PR+SD) (58 *vs* 37%, *P*=0.10) in favour of IL-2/HDC compared with IL-2 alone. Overall response rate was not significantly different between the two treatment groups.

The present analyses were based on 59 out of the 63 patients, receiving at least 80% of the scheduled drug dose in the first treatment course, in order to assess the *biological* effect of IL-2 with and without histamine in relation to baseline and on-treatment blood samples and tumour-tissue biopsies.

Patient characteristics were generally well balanced across treatment arms, although slightly more patients on the IL-2 arm had liver metastases and more patients on the IL-2/HDC arm were males (Table 2). These differences were not statistically significant (*P*=0.18 and *P*=0.054, respectively).

### Clinical outcome stratified for blood monocytes, neutrophils, NK and T cells

**Figure 1:**
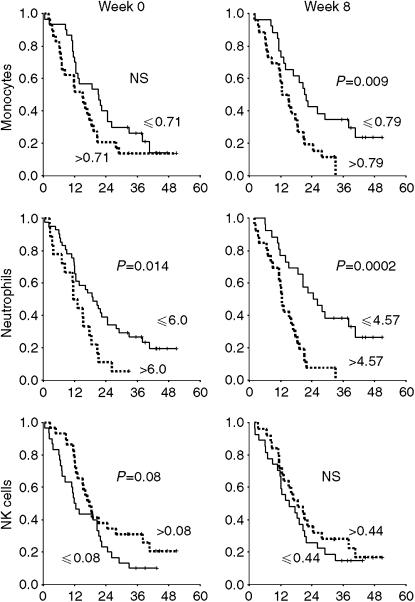
Peripheral blood monocytes, neutrophils and NK cells (CD16^+^56^+^) as prognostic factors for IL-2 based immunotherapy. High levels of monocytes and neutrophils were correlated with short survival.

**Figure 2:**
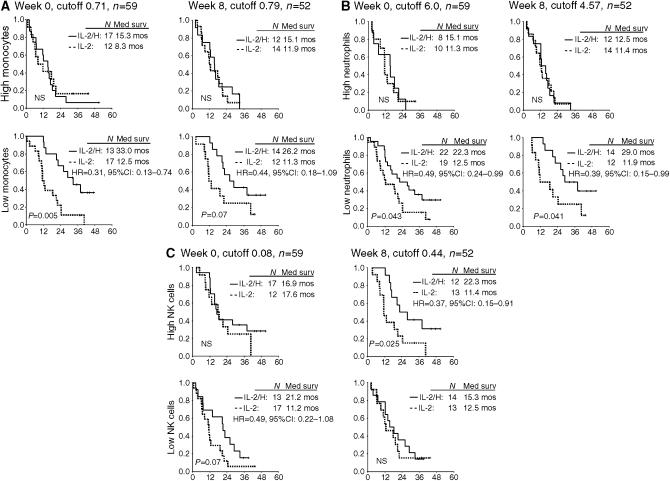
Peripheral blood neutrophils, monocytes and NK cells as prognostic factors stratified for treatment assignment. Kaplan–Meier plots at baseline and week 8 in patients with metastatic renal cell carcinoma treated with interleukin-2 and HDC (—) or IL-2 alone (- - - -) concerning survival for (**A**) monocytes (**B**) neutrophils and (**C**) NK cells (CD16^+^56^+^). IL-2/HDC treatment resulted in long-term survival in patients with low numbers of monocytes and neutrophils and patients with high number of NK cells in the peripheral blood. Vertical and horizontal axes, the survival probability and months of follow-up, respectively. Tick marks (∣) indicate last date of follow-up. The hazard ratio (HR) refers to IL-2/HDC relative to IL-2 alone. CI, confidence interval.

The prognostic impact of monocytes, neutrophils, NK and T-cells was analysed. As IL-2 treatment may induce considerably changes in the number and function of immune cells, baseline as well as on-treatment week 3 and week 8 values were correlated with treatment outcome. By keeping the on-treatment analyses de-linked from baseline, we evaluated the potential prognostic impact of high *vs* low numbers of immune cells, independently of previous measurements – with special emphasis on a possible histamine effect. Dichotomy of the variables was done at the median values. For baseline neutrophils, the median value (5.46) was close to the predefined cutoff level of 6.0 × 10^9^ l^−1^ (Lopez *et al*, 1996), which therefore was used as cutoff for the analyses. High levels of monocytes at week 8 (*P*=0.009) and neutrophils at baseline and at week 8 (*P*=0.014 and *P*=0.0002, respectively) were correlated with short survival (Figure 1). High levels of NK cells at baseline were correlated with long survival, however, of borderline significance (*P*=0.08). Baseline monocytes and NK cells at week 8 were not significantly correlated with survival. Further stratification by treatment assignment is shown in Figure 2. Patients with high number of monocytes and neutrophils in peripheral blood had a very poor survival, with apparently no benefit from either IL-2 alone or IL-2/HDC treatment. In contrast, survival was significantly improved in the IL-2/HDC group compared with the IL-2 alone group in patients with low number of baseline monocytes (*P*=0.005), low number of baseline neutrophils (*P*=0.043), low number of neutrophils at week 8 (*P*=0.041) and high number of NK cells at week 8 (*P*=0.025). The same trend, however, of only borderline significance, was seen in patients with low monocytes at week 8 (*P*=0.07) and low NK cells at week 0 (*P*=0.07) (Figure 2). Monocytes, neutrophils and NK cells, measured on week 3, showed the same trend as obtained at baseline and week 8 (data not shown).

We also evaluated blood lymphocyte subsets expressing CD4^+^, CD8^+^, CD56^+^, CD56^dim^, CD56^bright^, CD3^−^CD56^+^, CD56^+^CD57^+^ and CD57^+^. In general, the survival curves for these lymphocyte subsets were similar to the survival curves for the CD16^+^56^+^NK-cell. Thus, no significant survival differences were observed between ‘high’ and ‘low’ lymphocyte subsets. However, long-term survival was especially noted in patients treated with IL-2/HDC (data not shown).

### Circulating blood phagocytes

**Figure 3:**
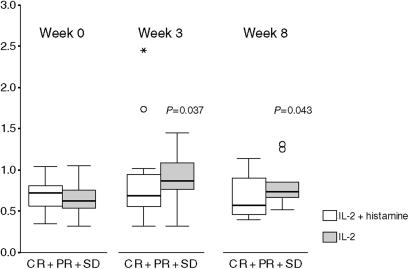
Potential histamine effect on circulating monocytes during IL-2-based treatment, assessed *in situ*. Treatment with IL-2/HDC (white boxes) resulted in unchanged number of circulating monocytes in patients with complete response (CR), partial responses (PR) and stable disease (SD) compared with baseline values, whereas treatment with IL-2-alone (black boxes) did not prevent a significant increased number compared with baseline. All patient with progressive disease (PD) had significantly increased monocyte numbers at weeks 3 and 8 compared with baseline, irrespective of the treatment group. Vertical and horizontal axes, 10^9^ cells l^−1^ and response to immunotherapy, respectively. The box plots represent the median (solid black line), the 25th and the 75th percentiles (boxed) and the 10th and the 90th percentiles (error bars). ‘O’ indicate outliers. ‘_^*^_’ indicate extremes.

The potential histamine effect on circulating phagocytes in peripheral blood was assessed *in situ* in blood samples obtained at baseline and during therapy at weeks 3 and 8. Non-PD patients (CR+PR+SD) treated with IL-2/HDC had unchanged number of circulating monocytes during treatment compared with baseline. In contrast, non-PD patients treated with IL-2 alone had significantly increased numbers at weeks 3 (*P*=0.037) and 8 (*P*=0.043) compared with baseline (Figure 3). Moreover, all PD patients had significantly increased monocyte numbers at weeks 3 and 8 compared with baseline, both for patients treated with IL-2/HDC (*P*=0.01 and *P*=0.008, respectively) and for patients treated with IL-2 alone (*P*=0.01 and *P*=0.05, respectively), data not shown. Circulating neutrophils were evaluated similarly; however, no statistically significant differences were found.

### Tumour-infiltrating immune cells

**Figure 4:**
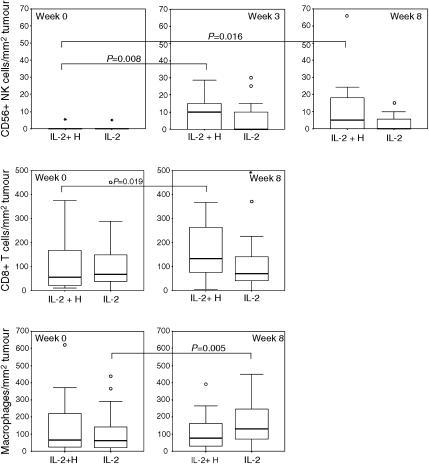
Potential histamine effect on intratumoral NK, T-cells and macrophages during IL-2 based treatment, assessed *in situ*. Treatment with IL-2/HDC resulted in significantly higher numbers of intratumoral NK cells and CD8^+^T cells and unchanged numbers of intratumoral macrophages during treatment compared with baseline. IL-2 alone did not prevent a significantly higher number of intratumoral macrophages but did not result in increased intratumoral NK or T cells compared with baseline. The box plots represent the median (solid black line), the 25th and the 75th percentiles (boxed) and the 10th and the 90th percentiles (error bars). ‘O’ indicate outliers. ‘_^*^_’ indicate extremes. Vertical and horizontal axes, the median number of cells mm^−2^ tumour tissue and therapy administered, respectively.

The potential histamine effect on the leucocyte subsets infiltrating the tumour tissue was assessed *in situ* in tumour core-needle biopsies obtained at baseline and during treatment at weeks 3 and 8. A total of 98 tumour biopsies were analysed (IL-2/HDC, *n*=47 and IL-2, *n*=51) (Figure 4). Patients treated with IL-2/HDC had significantly higher number of intratumoural CD56^+^ NK cells at weeks 3 (*P*=0.008) and 8 (*P*=0.016) and also higher number of intratumoural CD8^+^ T cells at week 8 (*P*=0.019) compared with baseline. In contrast, intratumoural macrophage numbers remained unchanged at weeks 3 and 8 compared with baseline (Figure 4). In comparison, patients treated with IL-2 alone had no increased intratumoural NK or T cells, but did show a significantly increased number of intratumoural macrophages at week 8 compared with baseline (*P*=0.005).

Intratumoural neutrophils showed no significant differences between treatment groups (data not shown)

### Functional assays

**Figure 5:**
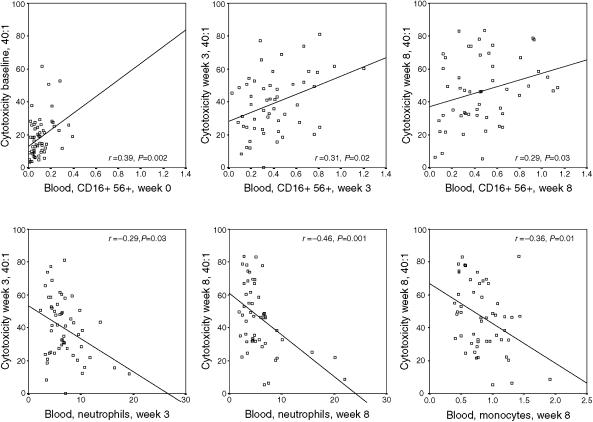
Correlation between *in vitro* cytotoxicity and NK cells, neutrophils and monocytes. A significantly positive correlation between blood CD16^+^CD56^+^ NK cells and cytotoxicity was observed. In contrast, there was a significantly negative correlation between blood neutrophils and cytotoxicity and also between blood monocytes and cytotoxicity.

An activation receptor for NK cells is CD16 (Herberman, 2002). Therefore, we assessed CD16^+^CD56^+^NK cells in peripheral blood as a functional assay. The number of CD16^+^56^+^NK cells increased median 350% (week 3) and 398% (week 8) compared with baseline values (*P*<0.001 and *P*<0.001, respectively), however, with no significant difference between the IL-2/HDC and the IL-2 alone group. In parallel, *in vitro* cytotoxicity increased median 121% (week 3) and 155% (week 8) compared with baseline values. There was a significantly positive correlation between blood CD16^+^56^+^ NK cells and cytotoxicity at baseline (Spearman's *r*=0.39, *P*=0.002), week 3 (*r*=0.31, *P*=0.02) and week 8 (*r*=0.29, *P*=0.03) (Figure 5). In contrast, there was a significantly negative correlation between blood neutrophils and cytotoxicity at week 3 (*r*=−0.29, *P*=0.03) and week 8 (*r*=−0.46, *P*=0.001) and also between blood monocytes and cytotoxicity at week 8 (*r*=−0.36, *P*=0.01) (Figure 5). The expression of the blood lymphocyte activation marker CD69 was significantly higher at week 8 compared with baseline in patients treated with IL-2/HDC (*P*=0.001), whereas no increase was observed in patients treated with IL-2 alone. CD3-zeta chain expression in blood and tumour did not show a statistically significant histamine effect compared with the effect of IL-2 alone.

## DISCUSSION

The results of the present biological part of our prospective randomised phase II trial provide evidence for circulating monocytes and neutrophils as negative prognostic factors for IL-2-based immunotherapy. Clearly, patients with high numbers of monocytes and neutrophils in peripheral blood had very poor survival, with apparently no impact of either IL-2-alone or IL-2 plus histamine treatment. Blood monocytes and neutrophils were negatively correlated with cytotoxicity, whereas blood NK cells were positively correlated with cytotoxicity, although not all differences achieved statistical significance, which may be due to the relatively small patient number. Thus, our data are highly supportive of the oxidative stress hypothesis formulated by Hellstrand *et al* (2002). Previously, three large studies of prognostic factors for IL-2 based immunotherapy in mRCC, including a total of 1422 patients, have all identified baseline-elevated neutrophils and biological signs of inflammation as poor prognostic factors (Lopez *et al*, 1996; Negrier *et al*, 2002; Atzpodien *et al*, 2003). Moreover, a reduction in blood monocytes during IL-2 treatment has been correlated with clinical response (Hermann *et al*, 1991). However, these studies were all purely descriptive with no ability to explain their findings. It is particularly noteworthy that the present study not only identifies baseline and on-treatment-elevated neutrophils and monocytes as poor prognostic factors, as we also did in a previous study (Donskov *et al*, 2004a), but offers an explanation of these findings by supporting the oxidative stress hypothesis. Our results therefore add to the understanding of how reactive oxygen-radicals influence the biological *in vivo* activity of immune cells during IL-2 based immunotherapy.

Chronic immune activation and chronic inflammation have long been suspected to be promoters of malignancy (Balkwill and Mantovani, 2001; Coussens and Werb, 2002). Among macrophage and neutrophils products, ROS may not only induce genomic instability (O'Byrne and Dalgleish, 2001), but also damage antitumour immune effector cells (Seaman *et al*, 1982; Hellstrand *et al*, 1994; Hansson *et al*, 1996). Intratumoural macrophages isolated from melanoma metastases inhibit NK-cell function by the release of ROS (Kono *et al*, 1996). Intratumoural NK and T cells isolated from mRCC show signs of oxidative damage (Finke *et al*, 1993; Tartour *et al*, 1995). IL-2 cannot activate NK cells *in vitro* in the presence of monocytes or macrophages (Hellstrand and Hermodsson, 1990). Based on these observations, it seems convincing that the observed survival differences for patients in the present study are caused by a phagocyte characteristic, namely the production of ROS, although we did not perform any direct measurements of ROS as purification methods and laboratory processes are difficult to perform without inducing stimulation in monocytes or neutrophils. Further investigation of the relation between phagocytes, NK cells, T cells and ROS in blood and tumour tissue is warranted, including direct measurements of apoptosis and ROS levels.

Our data support a novel treatment strategy involving the blocking of phagocyte-generated ROS by histamine, thereby protecting NK and T cells from apoptosis and, thus, synergising with IL-2 in inducing NK- and T-cell activation (Hellstrand and Hermodsson, 1990; Hellstrand *et al*, 1990; Asea *et al*, 1996; Hansson *et al*, 1999). Clearly, with regard to the ‘good guys’, treatment with IL-2/HDC resulted in significantly higher numbers of intratumoural NK and CD8^+^T cells during treatment compared with baseline. In contrast, treatment with IL-2 alone did not result in an increased number of intratumoural NK or T cells. Moreover, with regard to the ‘bad guys’, treatment with IL-2/HDC resulted in an unchanged number of circulating monocytes and intratumoural macrophages during treatment compared with baseline, whereas treatment with IL-2-alone did not prevent a significantly higher number of both circulating monocytes and intratumoural macrophages developing during treatment compared with baseline. It should be noted that low numbers of monocytes and neutrophils or high number of NK cells in peripheral blood were correlated with long-term survival. In these subgroups, a substantially increased survival rate was observed in patients treated with IL-2/HDC compared with patients treated with IL-2 alone. Thus, targeting H_2_O_2_ by histamine seems to enhance the antitumour activity of IL-2 *in situ* in a subgroup of patients. Recently, a superoxide dismutase mimetic, M40403, targeting superoxide (O_2_^•−^) was reported to enhance the antitumour action of IL-2 in mice models (Samlowski *et al*, 2003). As a result, developing and assessing drugs that block the generation of oxygen radicals as an adjunct to IL-2 is a viable therapeutic opportunity in renal cell cancer.

A randomised phase II trial of IL-2 with and without HDC in mRCC was run independently in Manchester, UK in parallel with the present trial (Donskov *et al*, 2005). The two studies had exactly the same clinical design and patient selection criteria. The outcome of the individual studies differed as the Danish study (*n*=63) showed a trend towards improved efficacy in favour of IL-2/HDC, whereas the UK study (*n*=41) was negative for all end points (Donskov *et al*, 2005). So, based on the randomised trials by themselves, no clear-cut effect of adding histamine to IL-2 was recognized. However, by the present biological analyses, a potential effect of histamine was clearly demonstrated, emphasising the value of accompanying clinical trials with blood and tumour tissue assessments. Indeed, the present assessment of the oxidative stress hypothesis in blood and tumour tissue is the first to establish a biological rationale – in humans – for the use of histamine in conjunction with IL-2 despite several clinical trials (Brune and Hellstrand, 1996; Mellqvist *et al*, 1999; Agarwala *et al*, 2002, 2004; Lurie *et al*, 2002; Schmidt *et al*, 2002; Donskov *et al*, 2002b). Previously, we have tested histamine in mRCC with *low-dose* IL-2 and interferon-alpha (IFN) (Donskov *et al*, 2002b) with no difference in treatment groups observed (Donskov *et al*, 2004a). However, the question of whether histamine might improve efficacy with higher doses of IL-2 formed the basis for the present randomised phase II study. Thus, in the present study, we have doubled the dose of IL-2 compared to that in our first IL-2/IFN/Histamine-study and the applied IL-2 dose can be considered to be an intermediate dose level.

Since its introduction to the clinic in 1985 (Rosenberg *et al*, 1985), IL-2 remains the only established immunotherapy approved by the US Food and Drug Administration for the treatment of metastatic melanoma and mRCC. However, despite 20 years of clinical trials, no combination therapy has proved better than IL-2 treatment alone in terms of long-term survival (Negrier *et al*, 1998; McDermott *et al*, 2005). Thus, cancer immunotherapy is most dependent on the application of advances in knowledge of basic science and its translation to the clinic. The present study indicates a direction for future clinical immunotherapy efforts.

In conclusion, our study provides evidence for circulating monocytes and neutrophils as powerful negative prognostic factors for IL-2-based immunotherapy and establishes a biological rationale for the potential use of histamine in conjunction with IL-2 in mRCC. However, patient numbers are too small to make definitive conclusions. Thus, a large confirmatory randomised phase III trial of IL-2 with and without histamine in mRCC appropriately stratified for monocytes and neutrophils in blood and tumour tissue is warranted.

## Figures and Tables

**Table 1:** Number of tumour biopsies and blood samples

**Week**	**0**	**3**	**8**
Number of patients on-treatment at the indicated time point	59	57	55
Number blood samples	59 (100%)	54 (95%)	52 (95%)
Number tumour biopsies	44 (75%)	29 (51%)	25 (45%)

Percentages indicate the number of samples obtained compared to the number of patients on-treatment at the indicated time point. The main reasons for not having a biopsy were withdrawal of consent and tumour necrosis. Not having a blood sample was due to logistic reasons.

**Table 2:** Baseline patient characteristics

	**IL-2 plus Histamine Study Group *N*=30**		**IL-2 alone Control Group *N*=29**	
	***N***	**%**	***N***	**%**
Median age, years (range)	55	(31–69)	60	(36–69)
Male	24	80	16	55
				
*Karnofsky performance status*				
100	16	53	12	41
90	10	33	13	45
80	2	7	2	7
70	2	7	2	7
				
Metastasis-free interval ⩽1 year	22	73	20	69
Baseline blood neutrophils				
Median (× 10^9^ l^−1^), (range)	5.43	(2.61–15.91)	5.54	(2.01–10.33)
Baseline blood monocytes				
Median (× 10^9 ^l^−1^), (range)	0.74	(0.35–1.51)	0.69	(0.32–1.11)
Baseline blood CD16^+^56^+^ NK-cells				
Median (× 10^9^ l^−1^), (range)	0.1	(0.02–0.36)	0.06	(0.01–0.39)
				
*Prior therapy*				
Nephrectomy	17	57	18	62
Excision of metastatic lesions	3	10	1	3
				
*Number of disease organ sites*				
1	2	7	3	10
2	7	23	7	24
3 or more	21	70	19	66
				
*Most common sites of disease*				
Primary kidney tumour	13	43	12	41
Local recurrence kidney bed	4	13	5	17
Lung/pleura	23	77	22	76
Lung metastasis alone	1	3	0	0
Lymph node	21	70	17	59
Liver	3	10	7	24
Bone	11	37	10	35
Soft tissue	5	17	4	14
				
*MSKCC prognostic criteria* a				
Favorable prognosis	6	20	5	17
Intermediate prognosis	20	63	18	62
Poor prognosis	4	13	6	21

a Memorial Sloan–Kettering Cancer Center (Motzer *et al*, 1999).

## References

[bib1] Agarwala SS, Glaspy J, O'Day SJ, Mitchell M, Gutheil J, Whitman E, Gonzalez R, Hersh E, Feun L, Belt R, Meyskens F, Hellstrand K, Wood D, Kirkwood JM, Gehlsen KR, Naredi P (2002) Results from a randomized phase III study comparing combined treatment with histamine dihydrochloride plus interleukin-2 versus interleukin-2 alone in patients with metastatic melanoma. J Clin Oncol 20: 125–133 11773161 10.1200/JCO.2002.20.1.125

[bib2] Agarwala SS, Hellstrand K, Gehlsen K, Naredi P (2004) Immunotherapy with histamine and interleukin 2 in malignant melanoma with liver metastasis. Cancer Immunol Immunother 53: 840–841 15127238 10.1007/s00262-004-0537-5 PMC11034204

[bib3] Asea A, Hermodsson S, Hellstrand K (1996) Histaminergic regulation of natural killer cell-mediated clearance of tumour cells in mice. Scand J Immunol 43: 9–15 8560202 10.1046/j.1365-3083.1996.d01-14.x

[bib4] Atzpodien J, Royston P, Wandert T, Reitz M (2003) Metastatic renal carcinoma comprehensive prognostic system. Br J Cancer 88: 348–353 12569375 10.1038/sj.bjc.6600768 PMC2747541

[bib5] Balkwill F, Mantovani A (2001) Inflammation and cancer: back to Virchow? Lancet 357: 539–545 11229684 10.1016/S0140-6736(00)04046-0

[bib6] Betten A, Dahlgren C, Hermodsson S, Hellstrand K (2003) Histamine inhibits neutrophil NADPH oxidase activity triggered by the lipoxin A4 receptor-specific peptide agonist Trp-Lys-Tyr-Met-Val-Met. Scand J Immunol 58: 321–326 12950678 10.1046/j.1365-3083.2003.01301.x

[bib7] Brune M, Hellstrand K (1996) Remission maintenance therapy with histamine and interleukin-2 in acute myelogenous leukaemia. Br J Haematol 92: 620–626 8616026 10.1046/j.1365-2141.1996.00389.x

[bib8] Chen JJ, Lin YC, Yao PL, Yuan A, Chen HY, Shun CT, Tsai MF, Chen CH, Yang PC (2005) Tumor-associated macrophages: the double-edged sword in cancer progression. J Clin Oncol 23: 953–964 15598976 10.1200/JCO.2005.12.172

[bib9] Coussens LM, Werb Z (2001) Inflammatory cells and cancer: think different!. J Exp Med 193: F23–F26 11257144 10.1084/jem.193.6.f23 PMC2193419

[bib10] Coussens LM, Werb Z (2002) Inflammation and cancer. Nature 420: 860–867 12490959 10.1038/nature01322 PMC2803035

[bib11] Donskov F, Bennedsgaard KM, Hokland M, Marcussen N, Fisker R, Madsen HH, Fode K, von der Maase H (2004a) Leukocyte orchestration in blood and tumour tissue following interleukin-2 based immunotherapy in metastatic renal cell carcinoma. Cancer Immunol Immunother 53: 729–739 15088127 10.1007/s00262-004-0525-9 PMC11032892

[bib12] Donskov F, Bennedsgaard KM, von der Maase H, Marcussen N, Fisker R, Jensen JJ, Naredi P, Hokland M (2002a) Intratumoural and peripheral blood lymphocyte subsets in patients with metastatic renal cell carcinoma undergoing interleukin-2 based immunotherapy: association to objective response and survival. Br J Cancer 87: 194–201 12107842 10.1038/sj.bjc.6600437 PMC2376103

[bib13] Donskov F, Middleton M, Fode K, Meldgaard P, Mansoor W, Lawrance J, Thatcher N, Nellemann H, von der Maase H (2005) Two randomised phase II trials of subcutaneous interleukin-2 and histamine dihydrochloride in patients with metastatic renal cell carcinoma. Br J Cancer 93: 757–762 16136045 10.1038/sj.bjc.6602768 PMC2361635

[bib14] Donskov F, von der Maase H, Henriksson R, Stierner U, Wersall P, Nellemann H, Hellstrand K, Engman K, Naredi P (2002b) Outpatient treatment with subcutaneous histamine dihydrochloride in combination with interleukin-2 and interferon-alpha in patients with metastatic renal cell carcinoma: results of an open single-armed multicentre phase II study. Ann Oncol 13: 441–449 11996477 10.1093/annonc/mdf049

[bib15] Donskov F, von der Maase H, Marcussen N, Hamilton-Dutoit S, Madsen HH, Jensen JJ, Hokland M (2004b) Fas ligand expression in metastatic renal cell carcinoma during interleukin-2 based immunotherapy: no *in vivo* effect of Fas ligand tumor counterattack. Clin Cancer Res 10: 7911–7916 15585624 10.1158/1078-0432.CCR-04-1111

[bib16] Finke JH, Zea AH, Stanley J, Longo DL, Mizoguchi H, Tubbs RR, Wiltrout RH, O'Shea JJ, Kudoh S, Klein E (1993) Loss of T-cell receptor zeta chain and p56lck in T-cells infiltrating human renal cell carcinoma. Cancer Res 53: 5613–5616 8242613

[bib17] Gundersen HJ, Bendtsen TF, Korbo L, Marcussen N, Moller A, Nielsen K, Nyengaard JR, Pakkenberg B, Sorensen FB, Vesterby A (1988) Some new, simple and efficient stereological methods and their use in pathological research and diagnosis. APMIS 96: 379–394 3288247 10.1111/j.1699-0463.1988.tb05320.x

[bib18] Hanahan D, Weinberg RA (2000) The hallmarks of cancer. Cell 100: 57–70 10647931 10.1016/s0092-8674(00)81683-9

[bib19] Hansson M, Asea A, Ersson U, Hermodsson S, Hellstrand K (1996) Induction of apoptosis in NK cells by monocyte-derived reactive oxygen metabolites. J Immunol 156: 42–47 8598491

[bib20] Hansson M, Hermodsson S, Brune M, Mellqvist UH, Naredi P, Betten A, Gehlsen KR, Hellstrand K (1999) Histamine protects T cells and natural killer cells against oxidative stress. J Interferon Cytokine Res 19: 1135–1144 10547153 10.1089/107999099313073

[bib21] Hellstrand K (2002) Histamine in cancer immunotherapy: a preclinical background. Semin Oncol 29: 35–40 10.1053/sonc.2002.33081 12068387

[bib22] Hellstrand K, Asea A, Dahlgren C, Hermodsson S (1994) Histaminergic regulation of NK cells. Role of monocyte-derived reactive oxygen metabolites. J Immunol 153: 4940–4947 7963557

[bib23] Hellstrand K, Asea A, Hermodsson S (1990) Role of histamine in natural killer cell-mediated resistance against tumor cells. J Immunol 145: 4365–4370 2147942

[bib24] Hellstrand K, Hermodsson S (1986) Histamine H2-receptor-mediated regulation of human natural killer cell activity. J Immunol 137: 656–660 3722819

[bib25] Hellstrand K, Hermodsson S (1990) Synergistic activation of human natural killer cell cytotoxicity by histamine and interleukin-2. Int Arch Allergy Appl Immunol 92: 379–389 2150668 10.1159/000235169

[bib26] Herberman RB (2002) Cancer immunotherapy with natural killer cells. Semin Oncol 29: 27–30 10.1053/sonc.2002.33079 12068385

[bib27] Hermann GG, Geertsen PF, von der Maase H, Zeuthen J (1991) Interleukin-2 dose, blood monocyte and CD25+ lymphocyte counts as predictors of clinical response to interleukin-2 therapy in patients with renal cell carcinoma. Cancer Immunol Immunother 34: 111–114 1760814 10.1007/BF01741344 PMC11038133

[bib28] Kono K, Salazar-Onfray F, Petersson M, Hansson J, Masucci G, Wasserman K, Nakazawa T, Anderson P, Kiessling R (1996) Hydrogen peroxide secreted by tumor-derived macrophages down-modulates signal-transducing zeta molecules and inhibits tumor-specific T cell- and natural killer cell-mediated cytotoxicity. Eur J Immunol 26: 1308–1313 8647210 10.1002/eji.1830260620

[bib29] Lin EY, Pollard JW (2004) Role of infiltrated leucocytes in tumour growth and spread. Br J Cancer 90: 2053–2058 15164120 10.1038/sj.bjc.6601705 PMC2410285

[bib30] Lopez HE, Kirchner H, Atzpodien J (1996) Interleukin-2 based home therapy of metastatic renal cell carcinoma: risks and benefits in 215 consecutive single institution patients. J Urol 155: 19–25 7490829

[bib31] Lurie Y, Nevens F, Aprosina ZG, Fedorova TA, Kalinin AV, Klimova EA, Ilan Y, Maevskaya MV, Warnes TW, Yuschuk ND, Hellstrand K, Gehlsen KR (2002) A multicentre, randomized study to evaluate the safety and efficacy of histamine dihydrochloride and interferon-alpha-2b for the treatment of chronic hepatitis C. J Viral Hepat 9: 346–353 12225329 10.1046/j.1365-2893.2002.00378.x

[bib32] Malmberg KJ (2004) Effective immunotherapy against cancer: a question of overcoming immune suppression and immune escape? Cancer Immunol Immunother 53: 879–892 15338206 10.1007/s00262-004-0577-x PMC11042482

[bib33] McDermott DF, Regan MM, Clark JI, Flaherty LE, Weiss GR, Logan TF, Kirkwood JM, Gordon MS, Sosman JA, Ernstoff MS, Tretter CP, Urba WJ, Smith JW, Margolin KA, Mier JW, Gollob JA, Dutcher JP, Atkins MB (2005) Randomized phase III trial of high-dose interleukin-2 versus subcutaneous interleukin-2 and interferon in patients with metastatic renal cell carcinoma. J Clin Oncol 23: 133–141 15625368 10.1200/JCO.2005.03.206

[bib34] Mellqvist UH, Wallhult E, Brune M, Jacobsson S, Hellstrand K (1999) Histamine dihydrochloride, interleukin-2 and interferon-alfa in multiple myeloma. Int J Immunother 15: 125–130

[bib35] Miller AB, Hoogstraten B, Staquet M, Winkler A (1981) Reporting results of cancer treatment. Cancer 47: 207–214 7459811 10.1002/1097-0142(19810101)47:1<207::aid-cncr2820470134>3.0.co;2-6

[bib36] Motzer RJ, Mazumdar M, Bacik J, Berg W, Amsterdam A, Ferrara J (1999) Survival and prognostic stratification of 670 patients with advanced renal cell carcinoma. J Clin Oncol 17: 2530–2540 10561319 10.1200/JCO.1999.17.8.2530

[bib37] Murdoch C, Giannoudis A, Lewis CE (2004) Mechanisms regulating the recruitment of macrophages into hypoxic areas of tumors and other ischemic tissues. Blood 104: 2224–2234 15231578 10.1182/blood-2004-03-1109

[bib38] Negrier S, Escudier B, Gomez F, Douillard JY, Ravaud A, Chevreau C, Buclon M, Perol D, Lasset C (2002) Prognostic factors of survival and rapid progression in 782 patients with metastatic renal carcinomas treated by cytokines: a report from the Groupe Francais d'Immunotherapie. Ann Oncol 13: 1460–1468 12196373 10.1093/annonc/mdf257

[bib39] Negrier S, Escudier B, Lasset C, Douillard JY, Savary J, Chevreau C, Ravaud A, Mercatello A, Peny J, Mousseau M, Philip T, Tursz T (1998) Recombinant human interleukin-2, recombinant human interferon alfa-2a, or both in metastatic renal-cell carcinoma. Groupe Francais d'Immunotherapie. N Engl J Med 338: 1272–1278 9562581 10.1056/NEJM199804303381805

[bib40] O'Byrne KJ, Dalgleish AG (2001) Chronic immune activation and inflammation as the cause of malignancy. Br J Cancer 85: 473–483 11506482 10.1054/bjoc.2001.1943 PMC2364095

[bib41] Pollard JW (2004) Tumour-educated macrophages promote tumour progression and metastasis. Nat Rev Cancer 4: 71–78 14708027 10.1038/nrc1256

[bib42] Rosenberg SA (2001) Progress in human tumour immunology and immunotherapy. Nature 411: 380–384 11357146 10.1038/35077246

[bib43] Rosenberg SA, Lotze MT, Muul LM, Leitman S, Chang AE, Ettinghausen SE, Matory YL, Skibber JM, Shiloni E, Vetto JT (1985) Observations on the systemic administration of autologous lymphokine-activated killer cells and recombinant interleukin-2 to patients with metastatic cancer. N Engl J Med 313: 1485–1492 3903508 10.1056/NEJM198512053132327

[bib44] Samlowski WE, Petersen R, Cuzzocrea S, Macarthur H, Burton D, McGregor JR, Salvemini D (2003) A nonpeptidyl mimic of superoxide dismutase, M40403, inhibits dose-limiting hypotension associated with interleukin-2 and increases its antitumor effects. Nat Med 9: 750–755 12730689 10.1038/nm874

[bib45] Schmidt H, Larsen S, Bastholt L, Fode K, Rytter C, von der Maase H (2002) A phase II study of outpatient subcutaneous histamine dihydrochloride, interleukin-2 and interferon-alpha in patients with metastatic melanoma. Ann Oncol 13: 1919–1924 12453861 10.1093/annonc/mdf325

[bib46] Seaman WE, Gindhart TD, Blackman MA, Dalal B, Talal N, Werb Z (1982) Suppression of natural killing *in vitro* by monocytes and polymorphonuclear leukocytes: requirement for reactive metabolites of oxygen. J Clin Invest 69: 876–888 7076851 10.1172/JCI110527 PMC370142

[bib47] Tartour E, Latour S, Mathiot C, Thiounn N, Mosseri V, Joyeux I, D'Enghien CD, Lee R, Debre B, Fridman WH (1995) Variable expression of CD3-zeta chain in tumor-infiltrating lymphocytes (TIL) derived from renal-cell carcinoma: relationship with TIL phenotype and function. Int J Cancer 63: 205–212 7591205 10.1002/ijc.2910630210

[bib48] Vakkila J, Lotze MT (2004) Inflammation and necrosis promote tumour growth. Nat Rev Immunol 4: 641–648 15286730 10.1038/nri1415

[bib49] Wyckoff J, Wang W, Lin EY, Wang Y, Pixley F, Stanley ER, Graf T, Pollard JW, Segall J, Condeelis J (2004) A paracrine loop between tumor cells and macrophages is required for tumor cell migration in mammary tumors. Cancer Res 64: 7022–7029 15466195 10.1158/0008-5472.CAN-04-1449

